# Uterine Hemorrhage

**Published:** 1846-04

**Authors:** 


*Uterine Hemorrhage.*—Few subjects connected with obstetrical science have created a greater sensation than the proposal of Drs. Radford and Simpson, to substitute extraction of the placenta, before the child, in certain cases of unavoidable hemorrhage, for the old-established rule of turning. It was not to be expected that a proceeding of so novel a character, and one so opposed to ordinary notions of correct practice, would be allowed to pass without criticism, and we accordingly find that it has met with the opposition of several accoucheurs of eminence, among whom we may mention Drs. Lee, Ashwell, &c. Of these, Dr. Lee has taken the most prominent part, having originated a controversy with Dr. Simpson, the heads of which we shall endeavor to reproduce.

In the first paper, published in the "Medical Gazette" of Sept 19, and containing the details of eight successful cases of placenta prævia, Dr. Lee condemns the new practice in the strongest terms, not only as opposed to the principles derived from the anatomical relations of the uterus, and placenta, and to the results of his own experience, but as not warranted even by the tables upon which Dr. Simpson has sought to establish the necessity for its adoption. This table Dr. Lee pronounces to be altogether fallacious, and adduces the evidence of Dr. Ramsbotham to show that Dr. Simpson has made the important error of mistaking the number of the infants lost for that of the mothers.

In reply to this communication, Dr. Simpson examines the objections of Dr. Lee seriatim. To the first of these, namely, that Dr. Lee sees no necessity for departing from the established rule of practice, he objects that if the result of the eight cases detailed by Dr. Lee was the expression of the real average of maternal danger, he should be the last to propose an alteration; but in order to show that such is not the case, he takes 61 cases which occurred to Drs. Lee and Ramsbotham, in which turning was adopted. Of this number, 24 mothers, or 1 in $2\frac{1}{2}$, were lost, a mortality which, as he observes, is equal to that of the Cæsarean operation. In answer to the accusation of Dr. Lee, that he (Dr. S.) advises extraction of the placenta in preference to turning, in all cases indiscriminately, Dr. Simpson replies that such is not the fact, but that he recommends the practice only under the following circumstances: In severe cases of unavoidable hemorrhage, complicated with a rigid and undilated os uteri, in which turning would be either impossible or hazardous; in most primiparæ; in unavoidable hemorrhage, with premature labor, and an undeveloped condition of the os and cervix; in placenta presentations with distorted pelvis; in cases where the extreme exhaustion of the patient forbids turning, and when the fœtus is ascertained to be dead, or is premature, and not viable. In support of these views of the necessity of the new proceeding, eleven cases are quoted from Dr. Lee's own practice, in which unavoidable hemorrhage was complicated with rigid and undilated os uteri. Of these eleven, three mothers only survived, and of these, two very nearly perished. Dr. Lee's third objection to extraction of the placenta, that it was not adopted by the older accoucheurs, is met by Dr. Simpson with the very obvious plea, that if any novel practice is to be condemned, simply because it was not thought or approved of by the ancients, there would be at once an end to all improvement, Dr. Simpson also demonstrates the error of Dr. Lee in asserting that Portal, one of the old authors referred to, never attempted to detach the placenta in any case, by quoting that author's own words, in which the fact of his having abstracted the placenta before the child is unequivocally expressed. Dr. Lee's fourth objection is one which apparently carries great weight, being this—that by the new practice the child must inevitably be sacrified. To this Dr. Simpson replies that, according to Dr. Lee's own showing, 65 per cent. of the infants perished under the operation of turning, in actual numbers 15 out of 23 in which the result is mentioned; while of 106 cases previously reported (Lond. and Edin. Monthly Journal, March, 1845), in which the placenta was spontaeously expelled before the child, 31 per cent. were saved, so that in this respect there is too little difference in mortality, to found an objection upon. "Besides," says Dr. Simpson, "the cases in which extraction of the placenta is to be advised, are exactly those in which the fœtus may be expected to be lost under any mode of treatment."

In answer to Dr. Lee's objection, upon the score of inaccuracy, Dr. Simpson refers the reader to a series of letters from Dr. Ramsbotham and Dr. Lee, which show that inaccuracies have crept into the statements of both parties, and that in this respect Dr. Lee is not less chargeable than his opponent.

The subsequent number of the same Journal contains an additional paper by Dr. Lee, in which 89 cases by Dr. Merriman, and 19 by Giffard, are tabulated. In the former of these there is evidently a mistake; 59 mothers out of 78, in which turning was adopted, being represented as having perished; while Dr. Merriman expressly states that he lost only 19; the numbers should clearly be reversed, but even this would make a maternal mortality of 1 in 4, not much short of that asserted by Dr. Simpson. In the 19 cases related by Giffard 5 died, also about 1 in 4, but these, Dr. Lee observes, perished from loss of blood prior to turning, and not in consequence of that operation.

It must be obvious, from the above imperfect abstract of this important controversy, that neither party is exactly in a condition to establish his position; statistical data, of *undoubted* accuracy, being absolutely necessary for so doing.—Looking, therefore, upon the question as one still undecided, we shall proceed to mention the opinions of other writers as to its feasibility, and to record the individual experience of those who have adopted the practice of Dr. Simpson.

Dr. Ashwell expresses his doubt of the correctness of Dr. Simpson's assertion, that the maternal deaths under the operation of turning in unavoidable hemorrhage are as high as 1 in 3. He states that in his own practice, he has lost only 2 out of 20; and that in the practice of the lying-in charity of Guy's Hospital, 2 cases in 14 only proved fatal. This writer also questions the fact upon which, as we have before said, Dr. Simpson bases the propriety of his operation, viz: that the blood in hemorrhage from placental presentation comes from the detached placenta. He shows satisfactorily that Dr. Lee is correct in denying the possibility of such an occurrence, as, in the first place, the decidual vessels are too small to allow of the terrific gushes of blood which are witnessed in this form of hemorrhage, and, moreover, that in consequence of the cellular structure of the placenta, its meshes become almost necessarily plugged by coagulated blood. As regards the new practice, Dr. Ashwell observes that it rarely requires to be employed, and never can be without risk to the mother, and certain death to the child. He thinks that in all cases in which the placenta could be safely removed, a little patience would also accomplish sufficient dilatation of the os uteri to admit of turning.

An opinion similar to the above is also expressed by Mr. Crowfoot, who has been fortunate enough not to lose a single patient in eleven cases of turning under unavoidable hemorrhage. Of the infants 4 perished. In two other cases, however, in which, from the quantity of blood lost, the fœtuses were evidently dead, Mr. Crowfoot admits that he should have advised extraction of the placenta had he been aware of its effect upon the hemorrhage. Mr. Newnham, of Farnham, likewise offers a somewhat qualified opposition to the practice, thinking it advisable in some cases where it may be necessary to diminish the size of the head in consequence of deformed pelvis; objecting to it as a general rule. It must, however, be remembered that neither Dr. Simpson nor Dr. Radford contends for its general operation.

The periodical press during the last six months contains several cases in which the new practice has been successfully adopted as regards the mother. The principal of these, to which the references will be found below, are as follows: Two cases by Messrs. Wilkinson and Tennent; one by Mr. Greenhow; one by Mr. Jones, of Llanfair; one by Mr. Parker of Bridgewater; one by Mr. Walker, of Chesterfield (in this case hemorrhage ceased as soon as the placenta was removed, but turning was subsequently necessary); two cases by Mr. Favell, of Sheffield, in which the placenta was spontaneously expelled without hemorrhage; one by Dr. Maclean; one by Mr. Hutchinson; one by by Mr. Hickings, in which he believes that the earlier abstraction of the placenta would have prevented the alarming hemorrhage which had occurred; and, lastly, one by Mr. Wells.

The subject of accidental uterine hemorrhage has been brought before the profession by Mr. Adams, of Banchory. The views of the writer as to the source of its hemorrhage are greatly at variance with generally received opinions, as will be seen from the following conclusions:

"1. That the doctrine generally taught, namely, that in floodings after delivery the blood escapes by exudation from the placental vessels of the uterus, is founded on a mistaken hypothesis regarding the construction of the placenta; while the symptoms of the case which were supposed to uphold the doctrine of uterine torpor, that is to say, the flaccidity of the womb when felt externally, and its distension and relaxation as discovered by the introduction of the hand, are to be regarded as the effects, and not the causes, of the flow of blood.

"2. That all the phenomena of the case are satisfactorily accounted for upon the supposition that the hemorrhage proceeds from lesions which are proved to be of no uncommon occurrence, about the uterus and perineum after delivery, but that analogy would lead to the conclusion that they sometimes proceed simply from violent action of the uterine exhalents.

"3. That there is no reason from analogy to suppose that blood would flow from an organ or member, merely because it is in a relaxed state, provided it had sustained no solution of continuity.

"4. That it has been demonstrably proved that contraction of the uterus is no safeguard against flooding, and that flooding is not necessarily the consequence of non-contraction after delivery.

"5. That the facts and circumstances connected with the Cæsarean operation, and the removal of the placenta in inertia of the womb, militate strongly against the established, opinion, that flooding is apt to arise from non-contraction, or sudden emptying of the womb.

"6. That, beyond all dispute, instrumental and precipitate deliveries are those in which floodings are of the most frequent occurrence; and it is well ascertained that lacerations are common in both these cases.

"7. That the introduction of the hand into the uterus, with the intention of stimulating the organ, or compressing the bleeding point, is a practice highly dangerous, tending to produce increased determination of blood to the part, syncope, and convulsions; and that the only circumstance which could justify the most gentle modification of this practice is the existence of a coagulum in the vagina or womb.

"8. That, agreeably to my own experience, laying the head low, exposing the patient to a current of cool air, applying cold cloths to the parts, with small doses of opium and gentle compression of the abdomen, are means which will rarely fail to arrest the flow of blood; but that in extreme cases it may be warrantable to try to ascertain the situation of the bleeding point, and secure it by surgical means.

"9. That, as far as can be ascertained, fatal results under this mild system of treatment in former ages were of much more unfrequent occurrence than they have been under the harsh mode of treatment pursued for sometime past.

"Finally, that the flooding which follows delivery has none of the characters of a passive hemorrhage, as has been generally taught of late, but is to be referred to the class of active hemorrhages, and treated upon the principles applicable to them."—*Ibid.*

